# Comparison of genetic variation of wild and farmed Bream (*Abramis brama orientalis; berg, 1905*) using microsatellite markers

**Published:** 2014-09

**Authors:** Zeinab Zeinab, Ali Shabany, Hamed Kolangi-Miandare

**Affiliations:** Fishery Faculty, Gorgan University of Agriculture Science and Natural Resources, Gorgan, Iran

**Keywords:** Microsatellite, *Abramis brama*, Polymorphism, Hardy-Weinberg equilibrium

## Abstract

Bream (*Abramis brama orientalis*) is one of the most commercially valuable fish in the Caspian Sea. The aim of this study was to compare levels of genetic polymorphism between wild and farmed Bream populations using seven microsatellite loci. Genetic diversity was investigated by studying samples collected from two regions; Chaboksar and the Artificial Propagation Center of Guilan province. Allele frequency was found to have declined in wild and cultured fish due to inbreeding and genetic drift. Significant population differentiation (F_st_) was observed between wild and farmed populations, which could be explained by the low number of alleles in two populations. Significant deviations from the Hardy-Weinberg equilibrium were found at more loci. Beyond the null alleles' hypothesis, heterozygote deficiency may have arisen due to inbreeding. Both populations showed lowest genetic diversity according to the number of alleles and genotypes per each locus. This approach was carried out for the first time and could provide information regarding the genetic variability of farmed and wild *abramis brama* fish using microsatellite markers. Results could be used for the management and conservation of artificial Bream propagation programs.

## INTRODUCTION

Bream (*Abramis brama orientalis*) is a commercially valuable food fish teleost (family: *Cyprinidae*, order: *Cypriniformis*) of the Caspian Sea. It is also found in the Anzali wetland [1]. An artificial propagation and farming program has been carried out in Iran for the conservation of this fish. Characterization of the genetic structure of *Abramis brama*, currently bred in the aquaculture industry can be used to select strains for brood stock development and management plans and to improve its genetic diversity by minimizing inbreeding.

The deteriorating genetic diversity of wild fish stocks is an important problem caused by human activities such as pollution, overfishing, habitat destruction and blockage of migration paths [2]. Normal genetic diversity enables environmental adaptation that could affect survival chances of a species or population; it is therefore, essential for the long-term survival of species [3]. It is also an essential component of artificial selection programs that aim to enhance ecological or economically important traits. 

Higher levels of allelic variation at microsatellite markers can help address questions related to genetic structure. Microsatellites consist of multiple copies of tandemly arranged simple sequence repeats (SSRs) that range in size from 1 to 6 base pairs [4, 5]. Microsatellite DNA markers represent a suitable tool for genetic tagging of wild brood stock as well as farmed populations [6-10]. Therefore, microsatellite analysis was used in the present study to (a) investigate the genetic diversity of wild and farmed *Abramis brama* fish populations to determine whether fish culture activities and artificial propagation programs have reduced genetic variation in farmed and wild populations, and (b) to analyze genetic relationships among the samples.

Research on the genetic variation of farmed and wild populations has been reported for several species [11-13]. Studies have demonstrated reduced genetic variability of farmed stocks in comparison to wild populations for masu salmon (*Oncorhynchus masou*) [14], rainbow trout (*Oncorhynchus mykiss*) [15], Atlantic salmon (*Salmosalar L*.) [16, 17], brown trout (*Salmo trutta* L.) [18] and the common carp (*cyprinus carpio*) [19, 20]. 

The aim of the present study was to evaluate and compare the genetic diversity and differentiation of wild and farmed Caspian Sea Bream using microsatellite loci as genetic markers. The results will have implications not only for future Bream *Abramis brama* farming and breeding programs, but also for the conservation of genetic resources of this species.

## MATERIALS AND METHODS

**Sample collection and DNA extraction:** A total of 60 samples were collected from two stations, Chaboksar costal water and the Artificial Propagation center of Guilan province. Total genomic DNA was extracted using standard phenol/chloroform protocols from fin pectoral tissues [21]. Approximately 100 mg tissue was treated with 25 μl proteinase K (10 mg/ml) and 50 μl sodium dodecyl sulfate (SDS) (10%) in a 500 μl Sodium Chloride-Tris- Ethylenediamine tetra acetic acid (STE) buffer (0.1 M NaCl, 0.05 M Tris and 0.01 M Na_2_EDTA, pH: 8.0) overnight at 37°C. After incubation, DNA was isolated by two phenol-chloroform (25 phenol: 24 chloroform: 1 isoamyl alcohol) steps followed by precipitation with cold absolute ethanol. The extracted DNA was then preserved at -20°C until used. 

**Molecular analysis:** In this study, seven microsatellite markers were amplified by polymerase chain reactions (PCR) using the following primers: (Rser10, Ic654, MFW_7,_ MFW_26, _Bl_2_-114, Bl_1 _-153, M_4_ ((Table 1). Initial denaturation was achieved at 94°C for 3 min followed by 30 denaturation cycles for 30 s at 94°C, 30 s at the respective annealing temperatures extending to 72°C for 1 min. The final step was extended to 3 min at 72°C. PCR products were separated using 8% polyacrylamide gels stained with silver nitrate [22].

**Table 1:** Characteristics of *Abramis* *brama* microsatellite loci used in this study

**Microsatellite loci**		**Primer Sequence**	**N**	**Size (bp)**	**Annealing (˚C)**
Bl2-114	F:ATCACTGCCATTTTATTA R:CTGCTCCGCTCTGTTCCA		16	140-260	52
Bl1-153	F:GCACAGCTCTAATCGGTCACT R:TATGGTCAAACACGGGTCAA		14	201-271	53
M4	F:ACCGGGCTTTAGGCTGTTGGTCA R:TGAGACACATCCCATCACTGCCTACG		9	100-200	59
Ic654	F:TGAGCCGACACTAGAAACAGAGC R:GACAAAGTGCAGGCACAGAATG		9	128-160	52
MFW7	F:TACTTTGCTCAGGACGGATGC R:ATCCCTGCACATGGCCACTC		10	160-208	61
MFW26	F:CCCTGAGATAGAAACCACTG R:CACCATGCTTGGATGCAAAAG		9	100-144	48
Rser10	F:TGCGTAATCGTGAAGCGGTG R:GCCACTAAAGCGCAGAAGCC		13	164-248	57

N: number of allele.

**Statistical analysis: **The number of alleles per each locus, observed heterozygosity (Ho), expected heterozygosity (He), the number of observed alleles (Na), the number of effective alleles (Ne), Hardy-Weinberg equilibrium (HWE) and F_st_ values were calculated by Genealex ver.6.5 Software [23]. PopGene version 1.31 software was used to determine genetic distance and similarity [24] and the phylogenetic relationships between populations [25].

## RESULTS AND DISCUSSION

In this study, the genetic diversity of wild and farmed populations of *Abramis brama* were investigated at seven microsatellite loci. TheBl_2_-114primer showed the maximum allele number (16) compared to other primers. No statistically significant difference was observed between the numbers of alleles per each locus (Table 2). The number of effective alleles varied from 4.678 for IC_654_ to 10.667 for Bl_2_-114, which was lower than the observed number of alleles in all populations. The observed (H_o_) and expected (H_e_) heterozygosity means for all samples were 0.30-1.00 and 0.55-0.90 respectively. In the wild fish samples, the mean for heterozygosity and expected heterozygosity values were 0.664 and 0.845, respectively. In the farmed samples, these values were 0.707 and 0.852, respectively, but the differences were not statistically significant (p>0.05).

**Table 2:** Genetic diversity parameters for seven microsatellite loci in *A. Brama*

Location		MFW7	MFW26	Bl1-153	Bl2-114	M4	Rser10	Ic654
Wild fish	Na	8	7	13	12	9	13	8
	Ne	5.67	4.52	10.27	9.41	6.66	8.33	4.67
	Ho	0.55	0.30	1.00	0.70	0.75	0.65	0.70
	He	0.82	0.77	0.90	0.89	0.85	0.88	0.87
	PHw	***	***	*	Ns	***	**	***
Culture fish	Na	10	7	13	14	13	9	9
	Ne	7.08	4.52	9.63	0.70	9.63	4.52	7.08
	Ho	0.90	0.30	0.85	0.89	0.85	0.30	0.90
	He	0.85	0.77	0.89	10.27	0.89	0.77	0.85
	PHw	***	*	Ns	Ns	Ns	Ns	Ns

**Note:** Na, number of observed alleles; Ne, number of effective alleles; Ho, observed heterozygosity; He, expected heterozygosity; PHW, Hardy-Weinberg probability test

* *P*<0.05,

** *P*<0.01,

*** *P*<0.001, n.s, non-significant

Significant deviations from the Hardy-Weinberg equilibrium (HWE) were found at the loci levels (Table 2). All seven loci were tested for deviation from the HWE. Nine out of 14 (7 loci × 2 populations) possible HWE tests were statistically significant (P<0.05). Population differentiation (F_ST_) was moderate with a statistically insignificant F_ST _value of 0.024 between the wild and farmed fish populations; however, R_ST_ value was significantly high (0.100) between the two populations. Genetic distances and similarities [24] computed between the wild and farmed fish populations were 0.383 and 0.682, respectively. The Unweight Pair Group Method with Arithmetic Mean (UPGMA) dendorogram drawn based on genetic distance data showed that these two populations were two distinctly different clades. F_is _values ranged from 0.029 (the locus Bl_1_-153) to 0.431 (the locus MFW_26_) for the two populations.

Genetic diversity is a major topic in aquaculture, especially in fish breeding and artificial propagation activities. Genetic differences between farmed and wild populations must be identified and quantified prior to any aquaculture activation if effective monitoring of gene flow is to occur. It is, therefore, important that adequate amounts of this kind of genetic information be available both for farmed and wild populations [26]. Heterozygosity is important for both wild and farmed populations because it provides a large spectrum of genotypes for adaptive responses to changing conditions. Moreover, heterozygous individuals are usually superior to less heterozygous ones in terms of economically important characteristics such as growth, fertility and disease resistance [27].

The results of the present study indicated that the average number of alleles per locus and the observed heterozygosity in farmed fish were10.714 and 0.707, respectively. For the wild fish, these numbers were 10 and 0.664, respectively, indicating no statistical significance between the farmed and wild populations (p>0.05). Allele loss and heterozygosity in the *Abramis brama* population may be intensified by bottlenecks and inbreeding. In the current study, the observed heterozygosity for 7 microsatellite loci was lower than the expected heterozygosity in the two populations. It must be noted that observed heterozygosity is not always greater at all loci in wild as compared to farmed populations. The results of this study are similar to those reported for other farmed species using microsatellites and other molecular markers [28, 29]. For the present study, expected heterozygosity values were found to be 0·77–0·90 for the wild populations and 0·80–0·90 for the farmed ones, but these differences were not significantly different (P>0.05).These results are comparable with those describing microsatellite analyses of wild and cultivated European populations of *S. aurata* [30]. Heterozygosity reduction was detected in farmed and wild populations, both showing the lowest genetic diversity in terms of range number of alleles and genotypes per locus. According to previous studies, such low heterozygosity rises due to inbreeding and shrinking population sizes [31].

Both populations under study showed deviation from the HWE, since nine out of fourteen (7 loci × 2 populations) possible tests for HWE were statistically significant (P<0.05). Significant deviations from Hardy-Weinberg expectations (HWE) were observed in the two populations (Table2). Genetic drift, inbreeding and divergent evolution are likely to be the causes for deviation from the H–W disequilibrium [32]. Several theories explain deviations from the HWE, including inbreeding, intra-population structure (Wahlund effect), non-random sampling, selection against heterozygotisity and fishing pressure [31, 33, 34]. Although selection forces in aquaculture activities could cause farmed populations to deviate from the HWE, another likely underlying mechanism is the mixing of families in these populations [35].

Genetic analysis of molecular variance (AMOVA) is a suitable criterion to assess population structure and determine the differentiation and genetic similarity between populations [36]. Analysis of pairwise genetic differentiation revealed that F_st_ values were small (Table 3). According to our obtained F_st_ index, the genetic diversity between the two populations was 2%. The F_st_ index mean was about 0.024, which represents the low differentiation between the two populations. According to Wright [37], F_st_ value of less than 0.05 indicates low differentiation among communities.

**Table 3:** Number of migrant,F_is_ and F_st_ index of seven microsatellite loci in two populations for *Abramis* *brama*in Iran

Loci	MFW_7_	MFW_26_	Bl_1_-153	Bl_2_-114	M_4_	Rser10	IC_654_
F_st_	0.038	0.007	0.015	0.016	0.039	0.015	0.016
F_is_	0.138	0.431	-0.029	0.139	0.170	0.158	0.221

**Figure 1:**
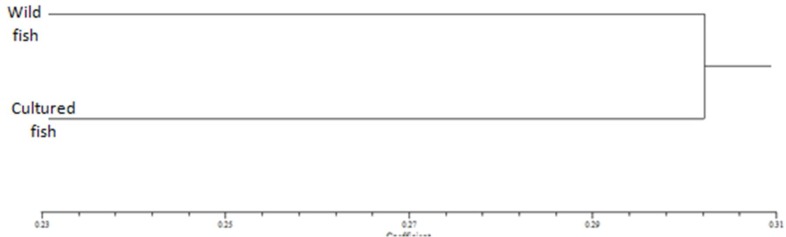
UPGMA dendrogram of wild and farmed *Abramis* *brama* samples

 In the present study, genetic distances among the two populations were small, that is, genetic distances and genetic similarity between the wild and farmed fish, as computed by the N_ei_ method [23], were 0.383 and 0.682, respectively. The UPGMA dendrogram which is based on genetic distance shows the two separate wild and farmed communities to have the genetic structure of *Abramis brama* [Fig. 1].

The results obtained from microsatellite markers showed high genetic diversity within and low diversity among populations. According to the analysis, it seems that the genetic diversity between farmed and wild fish is not statistically significant. Such situation is recommended for genetic diversity maintenance due to its important ecological roles. The results also indicate a tendency towards a reduction in variability within and between farmed and wild *Abramis brama* populations. The decline of genetic variability within the farmed and wild populations could be mainly due to the loss of (rare) alleles rather than reduced heterozygosity, and might have been caused by the relatively small number of breeders maintaining these strains. Such situations are typical for farmed fish due to the generally high fecundity of females. In the long run, this may lead to measurable inbreeding depressions such as reduced vitality and growth rate; therefore, inbreeding should be minimized by increasing the effective population size. According to recommendations by previous researchers [38], increases in inbreeding coefficients should not exceed 1% per generation. This requires an effective population size of at least 50 individuals (25 males and 25 females). In order to verify the trends of genetic changes observed in the present report, it is suggested that future genetic research on *Abramis brama* be directed towards larger numbers of wild and farmed populations with wider distributions.
